# A Message from the Surgeon General to the Medical Profession

**Published:** 1917-03

**Authors:** 


### A Message From the Surgeon General to the Medical Profession.

"Should the country ever be engaged in war, the Medical Department of the Army in calling reserve officers to the colors wishes to cause as little hardship and sacrifice to the reserve medical officers as may be consistent with the needs of the country. With this end in view, the Department desires that you bring to the attention of the profession at large the necessity of the city, county and State medical societies organizing for the purpose of taking care of the practices of the officers of the reserve who respond to a call for service. In England this plan has proven of great benefit. The idea of the Department is that the profession should organize upon a similar basis.

"For example, should Dr. Jones be called to the colors, the local medical society, through its members, would take charge of his practice during his absence. Upon relief from active duty his practice would be returned to him intact. Such a plan will cause no unnecessary hardship upon the officer responding to a call for service, while the absence of such plan would penalize the officer who gives his service to the country in a crisis. The Department appeals to the patriotism of the profession to protect the interest of those of the profession who may be called to duty in war."

